# Phenotyping of chronic pain in breast cancer survivors: an original study using the cancer pain phenotyping (CANPPHE) Network multidisciplinary international guidelines

**DOI:** 10.1007/s00520-024-08594-0

**Published:** 2024-05-27

**Authors:** Ismail Saracoglu, Meltem Isintas, Ali Turk, Laurence Leysen, Jo Nijs

**Affiliations:** 1 Department of Physiotherapy and Rehabilitation, Faculty of Health Sciences, Kutahya Health Sciences University, Kutahya, Turkey; 2 Department of Radiation Oncology, Faculty of Medicine, Kutahya Health Sciences University, Kutahya, Turkey; 3 Department of Senior Researcher Quality of Life, European Organisation for Research and Treatment of Cancer, Brussels, Belgium; 4 Pain in Motion Research Group (PAIN), Department of Physiotherapy, Human Physiology and Anatomy, Faculty of Physical Education & Physiotherapy, Vrije Universiteit Brussel, Brussels, Belgium; 5 Chronic Pain Rehabilitation, Department of Physical Medicine and Physiotherapy, University Hospital Brussels, Brussels, Belgium; 6 Department of Health and Rehabilitation, Unit of Physiotherapy, Institute of Neuroscience and Physiology, Sahlgrenska Academy, University of Gothenburg, Gothenburg, Sweden; 7 Center for Person-Centred Care (GPCC), Sahlgrenska Academy, University of Gothenburg, Gothenburg, Sweden

**Keywords:** Cancer pain, Central sensitization, Neuropathic pain, Nociceptive pain, Nociplastic pain, Mixed pain

## Abstract

**Purpose:**

The primary aim of this cross-sectional study is to examine the prevalence of pain phenotypes in breast cancer survivors (BCS). A secondary aim entails examining whether health related quality of life differs between the main pain phenotypes in BCS.

**Methods:**

BCS who experienced chronic pain were asked to complete the numeric pain rating scale for pain, Margolis pain diagram, and short form 36 (SF-36). Following administration of questionnaires and quantitative sensory examinations were applied. To determine the prevalence of the predominant type of pain, a recently proposed classification system by the Cancer Pain Phenotyping (CANPPHE) Network was used.

**Results:**

Of the 86 female participants, 19 (22.09%) had dominant neuropathic pain, 18 (20.93%) had dominant nociceptive pain and 14 (16.28%) had dominant nociplastic pain. 35 participants (40.70%) were classified as having mixed pain. One-way ANOVA revealed a significant difference between the four pain groups for the SF-36 general health (F = 3.205, *p* = 0.027), social functioning (F = 4.093, *p* = 0.009), and pain (F = 3.603, *p* = 0.017) subscale scores.

**Conclusion:**

This study found that pain in BCS was mostly of mixed phenotype, followed by predominantly neuropathic and nociplastic pain. Furthermore, it was found that, compared to BCS with predominant neuropathic and nociceptive pain, BCS with predominant nociplastic pain have lower health related quality of life in the areas of bodily pain and social functioning.

**Supplementary Information:**

The online version contains supplementary material available at 10.1007/s00520-024-08594-0.

## Background

According to the International Agency for Research on Cancer, breast cancer is the most common malignancy in women worldwide [1]. Survival is defined as the length of time a patient survives after being diagnosed with the disease. Survival rates in breast cancer have increased in recent years [2] and survivors often experience long-term symptoms that appear or persist after completing treatment. One of the most common persistent symptoms is pain in in breast cancer survivors (BCS). The high prevalence of pain (ranging from 11 to 46%) [3–5] in the growing population of cancer survivors is a major concern because pain is associated with poor health-related quality of life (HRQoL) and impaired daily functioning [6].

Despite the increasing number of studies in this field in recent years, the mechanisms underlying the pathogenesis of pain in cancer survivors remain unclear. Numerous patient-, treatment-, and cancer-related risk factors such as lymphedema, axillary lymph node dissection, chemotherapy, radiotherapy, hormone therapy, and smoking status have been shown to play an important role in the development of chronic pain in BCS [7]. New or worsening pain should be carefully investigated, as it may also be an indicator of recurrence or secondary malignancy [8].

As with all other chronic pain problems, patient-specific treatment for BCS is a rational and promising approach [9]. In the near future, precision medicine might allow patients to be classified into subgroups that differ in their susceptibility to, pathology, or prognosis of a particular disease or response to a particular treatment, enabling treatment to be tailored to individuals [10]. Mechanism-based classification of pain may be a critical step for implementing a precision medicine approach to the management of chronic pain in cancer survivors. Pain phenotypes in cancer survivors include the predominantly nociceptive, neuropathic, and nociplastic pain types defined by the International Association for the Study of Pain (IASP) [11] and mixed pain, which is a combination of these types [12]. In this context, Nijs et al. [13] developed the first mechanism-based classification system to distinguish the types of chronic pain seen in individuals with breast cancer. Using this classification system, Leysen et al. [14] concluded that the most common type of chronic pain in breast cancer patients was mixed pain (40.6%), followed by neuropathic pain (25.3%). However, the classification method used in their study does not include the current classification methods recommended for chronic pain phenotyping [15] and used the term central sensitization pain instead of nociplastic pain. Central sensitization is not part of the definition of nociplastic pain, but is the major underlying mechanism of nociplastic pain [16]. Additionally, central sensitization can also be seen in patients with nociceptive or neuropathic pain [17, 18]. Therefore, the ‘nociplastic pain’ term was introduced by the IASP in 2017. Furthermore, an updated stepwise classification system for post-cancer pain has since been proposed by the multidisciplinary international Cancer Pain Phenotyping (CANPPHE) Network in 2023 [19]. The advances in the field of cancer pain phenotyping create an important research question: do the previous findings regarding the prevalence of the 3 pain phenotypes in BCS, as previously documented [14], still stand with the CANPPHE pain phenotyping guidelines [19]. Therefore, the primary aim of this cross-sectional study is to examine the prevalence of pain phenotypes in BCS considering the recent advances in pain phenotyping. A secondary aim entails examining whether HRQoL differs between the main pain phenotypes in BCS (e.g., whether HRQoL differs between BCS with predominant neuropathic versus predominant nociplastic pain).

## Methods

### Study design

A cross-sectional, observational study was conducted to examine the 2 research aims. The study was approved by the non-interventional research ethics committee of Kutahya Health Sciences University (no: 2019/11–9) and prospectively registered at ClincalTrials.gov (NCT04219072).

### Participants

The following inclusion and exclusion criteria are listed below:

- Inclusion criteria
  - Participants had to meet the definition of cancer survivor set by the National Cancer Institute's Office of Cancer Survivorship [20]. According to this; the first criteria was set as having completed primary curative treatment for breast cancer at least 3 months before the study and being in full remission,
  - Experiencing pain or any somatosensory symptoms such as numbness, tingling, or burning within 1 year of breast cancer diagnosis (which possible indicates post-cancer pain),
  - Providing informed consent.
- Exclusion criteria
  - Reporting pain less than 3 points on the Numeric Pain Rating Scale [21]
  - Having other chronic disease,
  - Being diagnosed with a severe psychological or psychiatric disorder,
  - Having cognitive impairment, including dementia,
  - Being diagnosed with new neoplasm or metastasis,
  - Not completing any of the study assessments.

### Recruitment and setting

Participants were recruited by convenience sampling. Between June and October 2023, BCS presenting to the Radiation and Medical Oncology Center of Kutahya Health Sciences University Hospital were screened by an oncologist (A.T.) for eligibility at follow-up examinations and invited to participate in the study if eligible. All eligible participants were informed in advance about the procedures and evaluations to be performed in the study. After consent, all questionnaires were administered in person on the same day with the same test order as explained below, followed by scheduling the next appointment for conducting the quantitative sensory examination. All questionnaires with previous medical records were administered and recorded by a different clinician (M.I.).

### Assessments

#### Patient demographic/clinical data

Data regarding the participants’ age, type of cancer, prior cancer treatment, current medical condition, medical treatments, chronic comorbidities, and musculoskeletal disorders were first collected using a questionnaire (Supplement 1). For patients reporting previous or current musculoskeletal problems, we also reviewed their related imaging results for identifying any nociceptive sources. Musculoskeletal ultrasonography, MRI, computed tomography and radiography findings were reviewed for connective and bone tissue problems, EMG findings were reviewed for neural problems and Doppler ultrasonography findings were reviewed for lymphatic tissue problems. Clinical assessment (palpation and related physical tests) was also performed if deemed necessary by the same author (M.I.) who reviewed medical records.

#### Numeric pain rating scale

The Numeric Pain Rating Scale (NPRS) is used to assess the level of pain intensity [22]. Patients are asked to rate the severity of their pain on a scale of 0 to 10, where a value of “0” corresponds to no pain at all and a value of “10” corresponds to the worst pain imaginable. The NPRS was reported to have adequate clinimetric properties in patients with chronic cancer pain and high test-retest reliability (r = 0.80) [23].

#### Margolis pain diagram

The Margolis Pain Diagram consists of dorsal and ventral drawings of the body and is used to evaluate the location and distribution of pain [24]. Participants were asked to mark the place(s) where they felt pain for at least 24 hours during the previous 4 weeks*.* The test-retest reliability was reported high (r=0.85) in chronic pain patients [25].

#### Short form 36 (SF-36)

HRQoL was evaluated with the SF-36 scale, a generic self-report measure developed by the RAND Corporation [26]. The SF-36 consists of 36 items covering 8 domains: physical functioning (10 items), social functioning (2 items), role limitations due to physical problems (4 items), role limitations due to emotional problems (3 items), mental health (5 items), vitality/energy (4 items), bodily pain (2 items), and general health perceptions (5 items). Subscale scores range from 0 to 100, with higher scores representing better well-being in that domain. The Turkish adaptation, validity and reliability studies for the SF-36 in cancer patients were conducted by Pinar et al. [27]. They reported the test-retest reliability for the eight subscales of the SF-36 ranged between 0.81 and 0.94 [27].

#### Quantitative sensory examination

The quantitative sensory testing consisted of:

- Static tactile mechanical detection and the hot/cold detection
- Static and dynamic mechanical allodynia
- The vibration detection

All QST test were conducted by the same clinician (A.T.) at the same room temperature using the same test procedures and order for all participants. All tests were performed both in painful and pain-free areas. Responses were recorded as hypersensitive/allodynia, hyposensitive/loss of function, or normal (Table 1).

**Table 1:** Quantitative sensory examination

*Static Tactile Mechanical Detection*	Von Frey monofilaments (2 and 26 g) were used to evaluate static tactile mechanical detection [22, 23]. Each filament was applied four times and it was considered the loss of function when three stimulations produced no sensitive response No sensitivity to a 2 g monofilament was considered the loss of mechanical detection function. Similarly, no sensitivity to a 26 g monofilament was considered the loss of mechanical pain detection [23]
*Thermal Detection*	A metal object (coin) was used to determine hot/cold detection. A coin at room temp was applied to the skin to assess cold detection, and a coin held in the researcher’s pocket for 30 min was used to assess heat detection. Each test was applied four times and it was considered the loss of function when three application produced no sensitive response [24]
*Static Mechanical Allodynia*	Static mechanical allodynia (pressure/pain threshold) was evaluated using a digital algometer. Pressure of 4 kg was applied for 10 s and the pain responses (reported above 3 points in NPRS) were considered as static mechanical allodynia [9, 22]
*Dynamic Mechanical Allodynia*	A soft brush was used to evaluate dynamic mechanical allodynia. The brush was gently swept over the skin at a rate of 3–5 cm per second in a fixed direction and the responses were recorded. Pain responses (reported above 3 points in NPRS) were considered as dynamic mechanical allodynia [24]
*Vibration Detection*	A 128-Hz tuning fork was placed on certain bone protrusions (processus styloideus ulnae) and the responses were recorded [23]. The tuning fork was applied four times and it was considered the loss of function when three applications produced no sensitive response [24]

### Pain phenotyping procedure

**Fig. 1:**
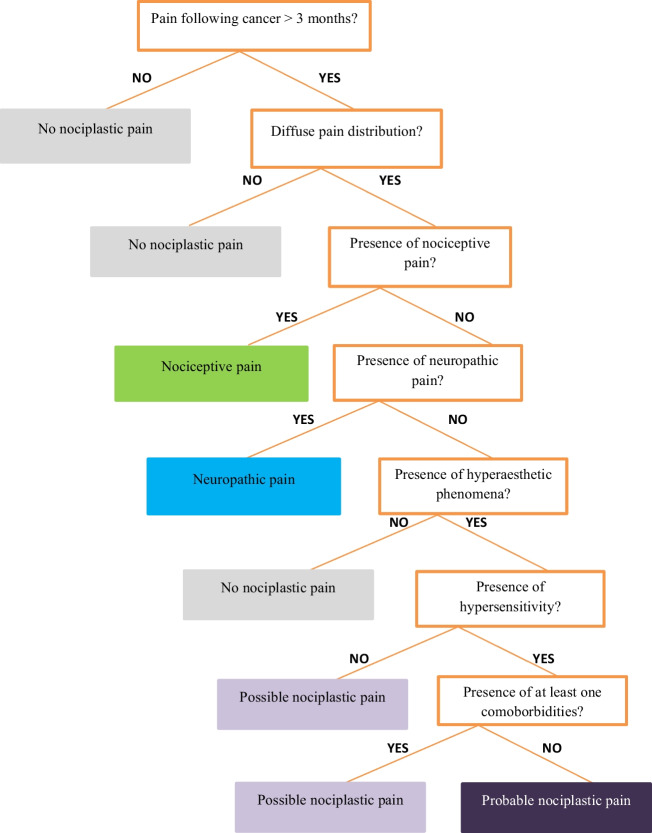
The seven-step clinical decision-making tree of the clinical criteria for nociplastic pain applied to post-cancer pain by CANPPHE

The stepwise classification procedure used in this study to identify nociceptive, neuropathic, and nociplastic pain was based on the IASP guideline developed by Kosek et al. in 2021 [11] and applied to post-cancer pain in 2023 by the international, multidisciplinary CANPPHE network [19]. The method consists of seven steps in total (Supplement 2). These steps investigate the following, in this specific order: pain duration, pain distribution, presence of nociceptive pain, presence of neuropathic pain, hypersensitivity phenomena, history of hypersensitivity, and presence of specific comorbidity. If there was overlap of more than one of the pain types (nociceptive, neuropathic, and nociplastic), the pain was classified as mixed type. The stepwise clinical algorithm is presented in Fig. 1.

All demographics, medical records, the results of physical examination and self-reported questionnaires summarized and reported as a case report by the researcher (M.I.). According to case reports, the classification process was carried out by the same researcher (I.S.) who did not conduct the patient assessments. The examiners (A.T. and M.I.) were also not involved in the pain phenotyping as described in Supplement 2. The pain phenotyping procedure as applied here, has been shown to have moderate to almost perfect intrarater reliability, weak to almost perfect interrarter reliability, and moderate criterion validity in non-cancer pain [28.]

### Sample size

The sample size for the study was calculated using the G*Power 3.1.9.7 program [29]. Based on the partial eta-square of 0.425 for the SF-36 general health subscale score in a study by Leysen et al. (Leysen et al., 2019), the number of samples required for one-way analysis of variance was calculated as 84 in total for a 5% margin of error (ɑ = 0.05) and 90% power (1-β = 0.90).

### Statistical analysis

The data were analyzed using SPSS version 17 package software. The Kolmogorov–Smirnov test was used as a normality test. As the data were normally distributed, one-way ANOVA was used to investigate differences in age, body mass index, pain duration, time since cancer diagnosis, pain severity, and HRQoL subscale scores according to pain phenotype. Significant F-tests were followed by Bonferroni's post hoc test. The Kruskal–Wallis test was used to examine differences between pain types for the following nominal variables: patient-related (pain medication use), disease-related (histological stage), and treatment-related (surgical method, chemotherapy, hormone therapy) risk factors. Significant results were further analyzed by pairwise comparisons using Mann–Whitney U test. For variables found to be significant in Bonferroni's post hoc and Mann–Whitney U tests, odds ratios were calculated to provide insight into the relationship between different pain phenotypes and patient-, disease-, and cancer treatment-related factors. A p- value of < 0.05 was considered statistically significant [30].

## Results

**Fig. 2:**
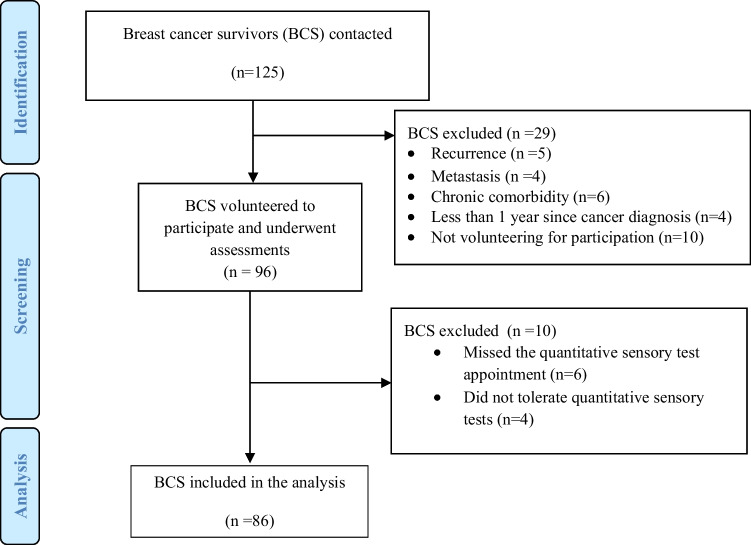
Flow diagram

From the 96 potentially eligible BCS, ten of them were excluded regarding eligibility criteria. Therefore, the study was completed with a sample of 86 patients. A flow chart illustrating the study process is presented in Fig. 2.

The mean age of the participants was 57.45 ± 5.44 years, all female, and the mean time since their breast cancer diagnosis was 48.09 ± 14.03 months. The participants’ mean NPRS score was 4.59 ± 1.36. The mean SF-36 subscale scores were 55.05 ± 17.68. All participants had undergone surgical treatment for breast cancer, mastectomy was performed in 58 patients (67.4%) and breast-conserving treatment was applied in the remaining 28 patients (32.60%). All participants received radiotherapy as part of cancer treatment (*n* = 86, 100%). Additionally, 60.5% (*n* = 52) received hormonal therapy and 52.3% (*n* = 45) received chemotherapy. Details on clinical characteristics are shown in Table 2.

**Table 2:** Demographic characteristics of breast cancer survivors by pain phenotype

	All (*n* = 86) *(Mean* ± *SD)*	Nociceptive (*n* = 18) *(Mean* ± *SD)*	Neuropathic (*n* = 19) *(Mean* ± *SD)*	Nociplastic (*n* = 14) *(Mean* ± *SD)*	Mixed (*n* = 35) *(Mean* ± *SD)*	*p*
Age (years)	57.45 ± 5.44	57.78 ± 6.20	55.11 ± 4.63	58.93 ± 2.92	57.97 ± 5.95	0.176
Body mass index (kg/m^2^)	26.51 ± 3.49	26.99 ± 4.18	26.93 ± 2.94	27.55 ± 3.57	25.63 ± 3.30	0.258
Months since cancer diagnosis	48.09 ± 14.03	50.89 ± 11.79	50.52 ± 16.05	46.86 ± 9.46	45.83 ± 16.06	0.535
Pain duration (months)	41.01 ± 15.19	41.00 ± 12.04	44.84 ± 18.37	40.72 ± 13.95	39.03 ± 15.48	0.621
Pain intensity (0–10)	4.59 ± 1.36	4.89 ± 1.02	4.00 ± 1.79	4.57 ± 1.45	4.77 ± 1.17	0.170
SF-36 general health	55.05 ± 17.68	63.61 ± 22.93	64.47 ± 22.66	49.29 ± 16.86	49.43 ± 15.89	0.027*
SF-36 physical functioning	64.82 ± 16.47	68.33 ± 14.55	60.52 ± 19.43	64.64 ± 20.14	65.43 ± 14.16	0.548
SF-36 social functioning	63.19 ± 24.60	80.14 ± 20.95^**+#**^	61.18 ± 26.96	57.14 ± 26.27^**+**^	58.00 ± 18.17^**#**^	0.009*
SF-36 mental health	61.13 ± 24.82	60.29 ± 20.55	70.37 ± 23.23	58.33 ± 20.78	58.11 ± 22.79	0.347
SF-36 energy/fatigue	53.04 ± 18.39	57.28 ± 15.81	59.95 ± 19.50	48.21 ± 16.00	49.06 ± 18.95	0.098
SF-36 bodily pain	52.55 ± 22.06	60.28 ± 19.85^**+**^	59.47 ± 24.30^#^	38.75 ± 20.72^**+**#^	50.35 ± 20.03	0.017*
SF-36 physical role limitations	65.04 ± 21.91	68.24 ± 25.32	62.29 ± 23.77	67.97 ± 19.95	80.66 ± 23.99	0.792
SF-36 emotional role limitations	65.37 ± 33.24	75.74 ± 20.58	57.88 ± 20.78	76.20 ± 24.21	59.91 ± 32.02	0.167
		*n (%)*	*n (%)*	*n (%)*	*n (%)*	
Histological grade						0.542
Grade 1		3 (16.7)	8 (42.1)	6 (42.9)	8 (22.9)	
Grade 2		13 (72.2)	7 (36.8)	5 (35.7)	18 (51.4)	
Grade 3		2 (11.1)	4 (21.1)	3 (21.4)	9 (25.7)	
Surgery methods						0.501
Breast conserving therapy		8 (44.4)	4 (21.1)	5 (35.7)	11 (31.4)	
Mastectomy		10 (55.6)	15 (78.9)	9 (64.3)	24 (68.6)	
Chemotherapy		7 (38.9)	10 (52.6)	8 (57.1)	20 (57.1)	0.628
Hormone therapy		7 (38.9) ^**$**^	9 (47.4)	13 (92.9) ^**$**^	23 (65.7)	0.010**
Pain medication		5 (27.8)	7 (36.8)	8 (57.1)	16 (45.7)	0.364

kg/m^2^: kilogram/ meter squared, SF-36: Short-form 36 health related quality of life questionnaire, n: Number of participants, SD: Standard deviation, *:*p* < 0.05 (One-way ANOVA), **:*p*-value < 0.05 (Kruskal–Wallis), ^+^:*p* < 0.05 (Bonferroni post-hoc procedure), ^#^:*p* < 0.05 (Bonferroni post-hoc procedure), ^$^:*p* < 0.05 (Mann–Whitney test)

**Fig. 3:**
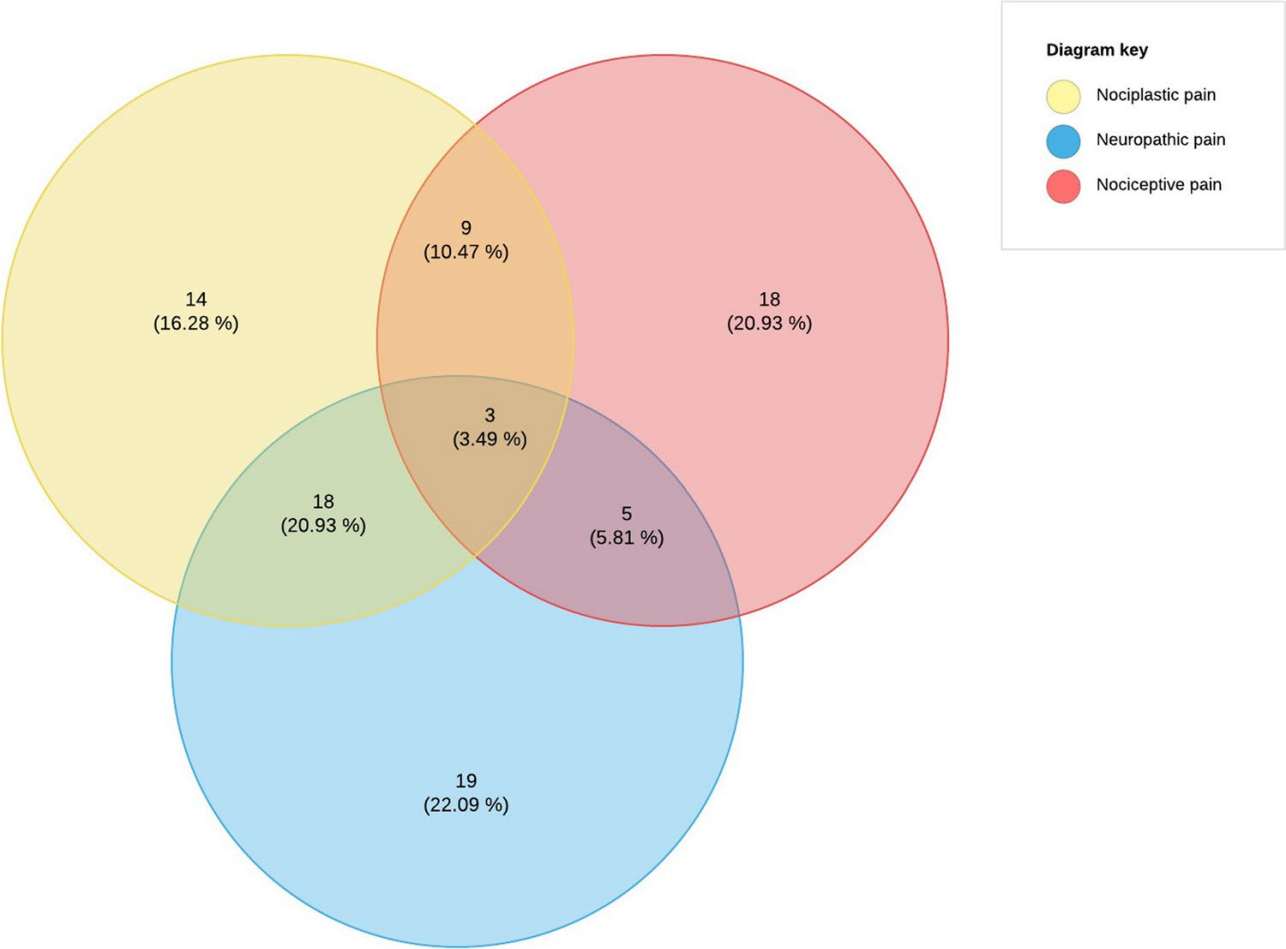
Prevalence of different pain phenotypes in 86 breast cancer survivors

Figure 3 shows the distribution of pain phenotypes in the study sample. Of the 86 participants, 19 (22.09%) had dominant neuropathic pain and 18 (20.93%) had dominant nociceptive pain. Another 14 (16.28%) were classified as dominant nociplastic pain. The remaining 35 participants (40.70%) were classified as having mixed pain. Five (5.81%) of these participants demonstrated mixed nociceptive/neuropathic pain, 9 (10.47%) mixed nociceptive/nociplastic pain, and 18 (20.93%) mixed neuropathic/nociplastic pain. Finally, mixed nociceptive/neuropathic/nociplastic pain was identified in 3 individuals (3.49%). In total, we detected a neuropathic pain component in 45 participants (52.3%), a nociceptive pain component in 35 participants (40.69%), and a nociplastic pain component in 44 participants (51.1%).

One-way ANOVA revealed a significant difference between the four pain groups for the SF-36 general health (F = 3.205, *p* = 0.027), social functioning (F = 4.093, *p* = 0.009), and pain (F = 3.603, *p* = 0.017) subscale scores (Table 2). The results of Bonferroni post hoc tests showed that the SF-36 social functioning scores were higher in participants with nociceptive pain than in those with nociplastic pain (mean difference: 22.99, 95% CI: -1.73 to 39.65, *p* = 0.041) and mixed pain (mean difference: 22.14, 95% CI: 4.13 to 40.14, *p* = 0.080). In addition, participants with nociplastic pain had lower bodily pain scores compared to those with nociceptive pain (mean difference: -21.53, 95% CI: -1.30 to -41.75, *p* = 0.031) and neuropathic pain (mean difference: -20.72, 95% CI: -0.75 to -40.96, *p* = 0.038). According to the Bonferroni post hoc analysis, no specific pain type differed significantly in the SF-36 general health subscale.

The Kruskal–Wallis test revealed a significant difference (*p* = 0.010) in the prevalence of hormone therapy among the groups (Table 2). The Mann–Whitney U tests demonstrated that this was attributable to the significant difference between the nociceptive and nociplastic pain groups (*p* = 0.020). The results of further analyses indicated that patients who received hormone therapy were 11 times more likely to have nociplastic pain than nociceptive pain compared to those who did not receive hormone therapy (odds ratio: 11.00, 95% CI: 1.36 to 88.60). Non-significant results were obtained for the remaining variables (*p* > 0.05).

## Discussion

This cross-sectional study examined the distribution of pain phenotypes in BCS with persistent pain and differences in HRQoL according to the different pain phenotypes. The results indicated that among the 86 participants, pain was predominantly neuropathic in 19 (22.09%), nociceptive in 18 (20.93%), and nociplastic in 14 patients (16.28%). Mixed pain was observed in the remaining 35 individuals (40.70%). Compared nociplastic and mixed pain, nociceptive pain was associated with higher HRQoL scores in the social functioning domain, whereas nociplastic pain was associated with lower HRQoL scores in the bodily pain domain compared to the nociceptive and neuropathic pain.

There are few studies regarding the prevalence of pain phenotypes in BCS. Leysen et al. [14] reported that neuropathic pain was more common than other predominant pain types in their study. Fuzier et al. [31] and Pereira et al. [32] also pointed out that neuropathic type pain may have a high prevalence in BCS. There might be several source of neuropathic pain in BSC. It might be caused by the cancer itself, neurotoxic agents used in chemotherapy [33], accidentally resection of the nerve during axillary surgery, or adhesion, inflammation, or fibrous tissue around the neural tissue [34, 35]. Similarly, excessive/prolonged radiotherapy can be an important reason for fibrosis and nerve compression [36]. The fact that all individuals in this study underwent surgery and radiotherapy can explain why neuropathic pain is the most common predominant pain phenotype. While our results similarly demonstrate that neuropathic pain was the most common predominant pain type, it is notable that mixed pain, which is an overlap of multiple pain phenotypes, was the most common phenotype overall. However, Leysen et al. [14] did not define mixed pain as a separate phenotype and did not report its prevalence, as they separately grouped all pain combinations that constitute mixed pain. It is also noteworthy that both in our study and the aforementioned study, neuropathic pain and nociplastic pain were the two most common components of the mixed pain phenotype [14]. Uncertainty regarding the mechanisms underlying mixed pain and the need to define and refine clinical criteria to identify patients with nociplastic pain [12] are important barriers to the recognition of this type of pain in BCS. Further studies are needed to determine the prevalence, mechanisms, and risk factors of mixed pain in BCS and other cancers.

A systematic review reported that young age, preoperative pain, intercostobrachial nerve injury during surgery, and radiotherapy, as well as insomnia due to frequent hospital visits, impaired cognitive function, and psychological morbidities such as anxiety and depression were among the factors most commonly associated with chronic postoperative pain in BCS [37]. Additionally, the adverse effects of radiotherapy, chemotherapy, and surgical treatment are common causes of neuropathic pain [38]. Central sensitization is one of the main mechanisms underlying nociplastic pain, another common type of pain identified in our study. Possible inflammation induced by cancer treatments, stress, anxiety, and sleep disorders are among the major risk factors for central sensitization and nociplastic pain [39]. An important finding of both the Leysen et al. study [14] and the current study is that individuals who received hormone therapy such as an aromatase inhibitor were more likely to experience nociplastic pain than those who did not. This suggests that the frequent history of hormone therapy in both studies may be one of the reasons for the high prevalence of nociplastic pain. Consistent with this idea, Joyce et al. [40] also reported that early discontinuation of hormone therapy by patients was an important risk factor for nociplastic pain. Developing both peripheral (e.g. in the joints) and central sensitization in response to aromatase inhibitors might be a major underlying mechanism of nociplastic and mixed pain [41]. Nevertheless, further research into the relationship between hormone therapies and central sensitization and nociplastic pain type are needed in the future.

Another interesting finding from our study was that BCS with nociplastic pain had worse HRQoL in the areas of bodily pain and social functioning. Previous studies have indicated that BCS have lower quality of life related to social and emotional functioning compared to the general population [42–44]. Factors such as catastrophizing [45], increased anxiety and worry [46], fear and avoidance behaviors [47], and inadequate coping strategies [48] have been shown to be strongly associated with disability in individuals with chronic pain and are among the common clinical characteristics of nociplastic pain. These factors may have had a greater impact on activities of daily living and social role participation in BCS. Based on the results of our study, we recommend a detailed assessment of psychosocial factors before planning pain management approaches in individuals exhibiting the nociplastic pain phenotype.

This research is among the pioneering studies to characterize pain in BCS according to the current terminology applied by the IASP and the latest guideline on post-cancer pain phenotyping from the international and multidisciplinary CANPPHE Network [19]. Our study may help to promote classification according to pain phenotype in the pain management of BCS and contribute to the implementation of precision medicine, an approach being widely adopted in the management of many diseases [49]. Other strengths of the study include its a priori registration, the standardization of the clinical examination, including administration of the self-reported measures and quantitative sensory testing, the blinding of the tester who conducted the quantitative sensory testing, and the use of different examiners for collecting and interpreting the data.

In addition to its strengths, this study has certain limitations. First of all, although the classification method used in this study has been shown to have moderate to almost perfect intrarater reliability, weak to almost perfect inter-rater reliability, and moderate criterion validity for differentiating pain phenotypes in non-cancer pain [28], studies examining the clinimetric properties of the international and multidisciplinary CANPPHE recommendations for pain phenotyping in cancer survivors are needed. The view of a second physician would have helped to examine the reliability of the findings reported here. In addition, recruitment for the study depended on the individuals’ willingness to participate, which may have introduced potential selection bias. The use of convenience sampling may also have led to under- or overrepresentation of certain groups in the sample. Besides, the comparison analysis of pain intensity between pain phenotypes should be interpreted carefully as NPRS is a doubtful interval scale. Lastly, any pain-related characteristics, especially etiological, beside the distinction between nociceptive/neuropathic/nociplastic or mixed pain was lacking in this study. Since the authors did not collected data systematically and they were not able to correctly identifying actual or threatened tissue damage for pain phenotyping process, the results needs to be interpreted carefully.

## Conclusion

In conclusion, by applying the current IASP terminology of nociplastic pain and the CANPPHE international and interdisciplinary pain phenotyping guidelines, this study found that the predominant pain types in BCS are neuropathic and nociplastic pain, but mixed type of pain appears to be the most prevalent phenotype in the BCS population. Furthermore, it was found that, compared to BCS with predominant neuropathic and nociceptive pain, BCS with predominant nociplastic pain have lower HRQoL in the areas of bodily pain and social functioning. Finally, hormone therapy was identified as a potential risk factor for nociplastic pain in BCS.

### Supplementary Information

Below is the link to the electronic supplementary material.

Supplementary file1 (DOCX 17 KB)

Supplementary file2 (DOCX 20 KB)

Supplementary file3 (DOCX 15 KB)

## Data Availability

The datasets generated and analyzed during the current study are available from the corresponding author on reasonable request.
