# Supplementary material for: Phenotyping of chronic pain in breast cancer survivors: an original study using the cancer pain phenotyping (CANPPHE) Network multidisciplinary international guidelines
Source: Support Care Cancer. 2024 May 27;32(6):383. doi: 10.1007/s00520-024-08594-0 (PMC11130012; doi:10.1007/s00520-024-08594-0)
Supplement: Supplementary file 1 — Supplementary file1 (DOCX 17 KB) [file 520_2024_8594_MOESM1_ESM.docx]

**Supplement 1. Questionnaire**

- Name:
- Date of birth:
- Weight: Height: BMI:
- When did your pain start (duration)?
- When did you receive your cancer diagnosis?
- Which histological grade of cancer were you diagnosed with?
- What treatments did you receive for cancer? (mark all that apply)

○ Surgery

○ Chemotherapy

○ Radiotherapy

- If you had surgery, what type of surgery was performed?
- Has the cancer spread (metastasis)?
- Has the cancer returned (recurrence)?
- Are you taking any medication or still receiving treatment for your cancer? If so, please indicate which medication(s) you take:

○ I do not use any medications

○ Pain killers

○ Cardiovascular medication

○ Diabetes medication

○ Hormonal drugs

○ Osteoporosis

○ Other
