# Supplementary material for: Phenotyping of chronic pain in breast cancer survivors: an original study using the cancer pain phenotyping (CANPPHE) Network multidisciplinary international guidelines
Source: Support Care Cancer. 2024 May 27;32(6):383. doi: 10.1007/s00520-024-08594-0 (PMC11130012; doi:10.1007/s00520-024-08594-0)
Supplement: Supplementary file 2 — Supplementary file2 (DOCX 20 KB) [file 520_2024_8594_MOESM2_ESM.docx]

**Supplement 2: CANPHEE stepwise grading system for pain phenotyping after cancer**

***Step 1***

The first step in applying the IASP clinical criteria for nociplastic pain requires that patients report pain of at least 3 months’ duration. According to the IASP clinical criteria, nociplastic pain can only be considered in patients having chronic pain.

***Step 2***

In addition to having chronic pain, patients must also report a regional, multifocal, or widespread, rather than discrete pain distribution to be clinically classified as having nociplastic pain. A thorough assessment and interpretation of self-reported pain distribution using body charts, in light of the identified possible sources of nociception and neuropathy, is required. Pain drawings were used to assess pain.

***Step 3***

The third mandatory criterion for nociplastic pain is that cancer survivors should report pain that cannot entirely be explained by nociceptive pain mechanisms. This includes either identifying or refuting nociceptive pain (including inflammatory pain) as the dominant post-cancer pain phenotype. It is appropriate here to assess the severity of the injury, pathology, and objective dysfunction capable of generating nociceptive input. This includes imaging techniques for identifying nociceptive sources in cancer survivors (e.g. musculoskeletal ultrasonography, radiography, MRI, and CT), but also an in-depth patient interview and clinical/behavioral assessment (including palpation, inspection, and physical testing including quantitative sensory testing). When nociceptive mechanisms are considered to be entirely responsible for the postcancer pain, the pain should be classified as ***nociceptive post-cancer pain***. In cases where nociceptive pain was not entirely responsible for the pain experience, the clinician continued to step 4.

***Step 4***

As with nociceptive pain, the fourth mandatory criterion for nociplastic post-cancer pain is that cancer survivors report pain that cannot entirely be explained by neuropathic pain mechanisms. This includes either identifying or refuting neuropathic pain as the dominant post-cancer pain phenotype. Researchers relied on the current guideline for classification of neuropathic pain, the IASP neuropathic pain special interest group guidelines on neuropathic pain assessment, and considered the results of quantitative sensory assessment. The neuropathic pain criteria specify that a lesion or disease of the central or peripheral nervous system is identifiable and that pain is limited to a ‘neuroanatomically plausible’ distribution. Hence, diagnostic procedures confirming or refuting the nervous system lesion or disease are mandatory for diagnosing neuropathic pain. When neuropathic pain was considered to be entirely responsible, the pain should be classified as ***neuropathic post-cancer pain***. In cases where neuropathic pain was not exclusively responsible for the post-cancer pain, the researchers continued to step 5. Moreover, the presence of neuropathic pain does not exclude the coexistence of nociplastic pain. Therefore, if neuropathic pain was present but was not considered entirely responsible for the pain, the clinicians continued to step 5.

***Step 5***

Step 5 includes screening for clinical signs of pain hypersensitivity that are at least present in the region of pain. This step entailed clinical examination of allodynia (hot, cold, static and dynamic) defined in quantitative sensory assessment. Immediate pain responses to any of these allodynia tests were considered a positive allodynia examination, and hence fulfilled this criterion. If the five requirements of the first five steps were met, the patient was classified as having ***possible nociplastic pain***, and researchers proceeded to step 6 to examine whether the likelihood of nociplastic pain can be increased to probable nociplastic pain.

***Step 6***

Step 6 involved examining whether the cancer survivor presents with a history of hypersensitivity in the region of pain. This was assessed by questioning the patient about sensitivity to touch, movement, pressure, or heat/cold.

***Step 7***

The final step involved screening for comorbidities in breast cancer survivors. This criterion was met if any of the following comorbidities were present: increased sensitivity to sound, light, and/or odors; sleep disturbance with frequent nocturnal awakening; fatigue; or cognitive problems. Similar to step 6, screening for these comorbidities was done during the patient interview and history. In addition, the central sensitization inventory (item numbers 12, 13, 17, and 20 questioning sleep, concentration, energy level, and hypersensitivity to odors) was considered when screening for these morbidities. Post-cancer pain was classified as ***probable nociplastic pain*** when cancer survivors fulfilled the criteria of steps 1-5, the patient presented with a history of pain hypersensitivity in the region of pain (step 6), and at least one of the defined comorbidities was present (step 7)*.*
