# Supplementary material for: Phenotyping of chronic pain in breast cancer survivors: an original study using the cancer pain phenotyping (CANPPHE) Network multidisciplinary international guidelines
Source: Support Care Cancer. 2024 May 27;32(6):383. doi: 10.1007/s00520-024-08594-0 (PMC11130012; doi:10.1007/s00520-024-08594-0)
Supplement: Supplementary file 3 — Supplementary file3 (DOCX 15 KB) [file 520_2024_8594_MOESM3_ESM.docx]

**Supplement 3. The results of quantitative sensory examinations**

|  | *Normal*  *n ( % )* | *Hypersensitivity/allodynia*  *n (% )* | *Hyposensitivity/loss of function*  *n (% )* |
| --- | --- | --- | --- |
| ***Static tactile mechanical detection*** | 31 (36) | 34 (39.5) | 21 (24.4) |
| ***Dynamic mechanical*** | 30 (34.9) | 37 (43) | 19 (22.1) |
| ***Hot detection*** | 36 (41.9) | 29 (33.7) | 21 (24.4) |
| ***Cold detection*** | 32 (37.2) | 34 (39.5) | 20 (23.3) |
| ***Vibration detection*** | 55 (63.9) | 14 (16.3) | 17 (19.8) |

n: Number of participants, %: Percentage
